# Heart rate variability helps to distinguish the intensity of menopausal symptoms: A prospective, observational and transversal study

**DOI:** 10.1371/journal.pone.0225866

**Published:** 2020-01-15

**Authors:** Patrícia Merly Martinelli, Isabel Cristina Esposito Sorpreso, Rodrigo Daminello Raimundo, Osvaldo de Souza Leal Junior, Juliana Zangirolami-Raimundo, Marcos Venicius Malveira de Lima, Andrés Pérez-Riera, Valdelias Xavier Pereira, Khalifa Elmusharaf, Vitor E. Valenti, Luiz Carlos de Abreu

**Affiliations:** 1 Laboratório de Delineamento de Estudos e Escrita Científica, Centro Universitário Saúde ABC, Santo André, São Paulo, Brazil; 2 Laboratório de Delineamento de Estudos e Escrita Científica do Laboratório de Práticas Cientíticas, Centro Universitário Uninorte, Acre, Brazil; 3 Disciplina de Ginecologia, Faculdade de Medicina, Universidade de São Paulo (USP), São Paulo, São Paulo, Brazil; 4 Graduate Entry Medical School, University of Limerick, Limerick, Ireland; 5 Centro de Estudos do Sistema Nervoso Autônomo (CESNA), UNESP, Marilia, SP, Brazil; All India Institute of Medical Sciences, INDIA

## Abstract

Heart Rate Variability (HRV) represents cardiac autonomic behavior and have been related to menopausal symptoms, mainly vasomotor symptoms and their imbalance to cardiovascular risk. It is not clear in the literature which index represents this imbalance and what is their involvement with the menopausal state. The aim of this study was to evaluate HRV in menopausal transition and post-menopausal symptoms with different intensities. This cross-sectional study was conducted in Rio Branco, State of Acre, Brazil from October 2016 to July 2017. We used Kupperman-Blatt Menopausal Index (KMI) to measure menopausal symptoms intensity. HRV analysis was performed based on the guidelines of the European Society of Cardiology and the North American Society of Pacing and Electrophysiology. HRV is lower in the group with moderate/intense menopausal symptoms compared to mild symptoms. (RMSSD: p = 0.005, Cohen’s d = 0.53, pNN50: p = 0.0004, Cohen’s d = 0.68; HF: p = 0.024, Cohen’s d = 0.44). There was association between HRV and KMI (RMSSD: r = -1.248, p = 0.004; and pNN50: r = -0.615, p: 0.029) in inverse relation to the intensity of vasomotor symptoms in women in TM. In conclusion, HRV was able to distinguish menopausal symptoms, indicating reduced vagal control in women with more intense symptoms.

## Introduction

The transition to menopause (TM) and postmenopausal (PM) comprise a life span related to biological, psychological and social changes that occur in the reproductive and non-reproductive period of women. It is characterized by the presence of menopausal symptoms and repercussions on different organs and systems, including cardiac autonomic regulation [1–4].

The main menopause symptoms comprises vasomotor (hot flushes), which arise from physiological hypoestrogenism due to progressive ovarian failure. These symptoms have been related to the action of the sympathetic and parasympathetic nervous systems [5]. In this sense, heart rate (HR) variability (HRV) is a non-invasive method that evaluates the autonomic nervous system (ANS) function used as a marker of autonomic homeostasis and considered a prognostic tool for cardiovascular morbidity and mortality [6–11].

Hypoestrogenism promotes modifications in the autonomic control of HR, inducing changes in sympathetic and vagal regulation of the heart rhythm [12, 13]. Akiyoshi et al. [12] demonstrated that cardiac parasympathetic function is reduced in TM and PM, which are associated to aging and hormone level. Freedman et al. [14] and Neufeld et al. [15] demonstrated that vasomotor symptoms are related with sympathetic predominance in the PM period.

Hot flushes, a major expression of vasomotor symptoms, are related to cardiovascular risks and reductions in cardiac vagal control analyzed through HRV, which increases cardiovascular morbidity and mortality [13, 16–20]. Thurston et al. [16, 17] and De Zambotti et al. [13] supported the reduction of vagal activity associated with hot flushes, regardless of the life span, reinforcing the development of cardiovascular complications.

Although it was documented autonomic dysfunction in the postmenopausal period via HRV, it is not clear whether this method is able to distinguish the symptoms intensities. This information would be extremely relevant for the medical team to better plan the treatment. Therefore, we aimed to evaluate HRV in menopausal transition and post-menopausal symptoms with different intensities.

## Methods

### STROBE guidelines

Our investigation follows instructions from the STROBE (STrengthening the Reporting of OBservational studies in Epidemiology) guidelines. Our study provides information of the study setting, design, variables, participants, description of potential sources of bias, data sources, measurement, quantitative variables description, and statistical methods.

#### Population study and eligibility criteria

The study population consisted of women who attended the first consultation over 40 years old and were divided into two groups according to their reproductive and non-reproductive period: 1) the menopausal transition group (TM, n = 71) was characterized by irregular menstrual cycle (absence of at least two menstrual cycles in 6 months) and presence of menopausal symptoms; 2) postmenopausal group (PM, n = 38), characterized by absence of menstrual cycle for 12 months, as defined by the North American Menopause Society [21, 22].

We did not include women under antidepressants, angiotensin converting enzyme (ACE) inhibitors, beta-blockers treatment, with fever, cardiomyopathies, decompensated heart failure, under hormone therapy, abnormal genital bleeding of unknown cause, hysterectomized, women with no precisely menopause determination, volunteers with any type of alteration in the ECG tracing (arrhythmias, below and / or above ST segment elevation), HR recording with less than 95% sinus and volunteers who refused to sign the free and informed consent form.

#### Ethical approval and informed consent

The study was approved by the Research Ethics Committee in Research of School of Medicine of ABC via Brazilian Platform (http://plataformabrasil.saude.gov.br/login.jsf) (Number 1.748.393 / 2016). Informed consent was provided to all participants. A statement from the Committee stated that the study were performed in accordance with the 466/2012 resolution of the National Health Council of December 12^nd^ 2012.

#### Study design and setting

This is a prospective, observational and cross-sectional study performed at Basic Health Units Mariano Gonzaga and Maximo Diogo Magalhães, Municipal Secretary of Health, in Rio Branco, State of Acre, Brazil from October 2016 to July 2017.

#### Bias

We completed all protocols under the same environments to address potential sources of bias. HR recording was performed at the same time of the day (between 8:00 and 12:00 am) to standardize circadian influences, in a room with temperature between 22°C and 25°C The subjects were recommended to avoid drinking coffee and other autonomic stimulants 24 hours before the data collection and to maintain an empty bladder during HR recording.

In order to characterize the sample, reduce the unpredictability of the variables, improve the reproducibility and physiological interpretation we measured age, mass (kg), height (m), systolic (mmHg) and diastolic arterial pressure (mmHg), abdominal circumferences (cm) and body mass index (BMI).

#### Initial assessment and experimental protocols

Firstly, all the subjects answered the Kupperman-Blatt Menopausal Index (KMI). Subsequently, we measured baseline arterial blood pressure and HR for 20 minutes in dorsal decubitus under spontaneous breathing.

### Variables, data sources and outcome measures

#### HRV analysis

RR intervals were recorded through the portable RS800CX monitor with a sampling rate of 1 kHz. The RR intervals were then transferred to the Polar Precision Performance program (v.3.0, Polar Electro, Finland). The Polar transmitter detects heart beats in the left ventricular muscle and transmit the signal to the computer through a wireless technology. The software allowed the visualization and the removal of RR interval file in “txt” format.

We performed HRV analysis based on directives from the Task Force guidelines [6]. Specific details of HRV analysis have been previously documented [23, 24].

HRV was analyzed in the time (RMSSD: root-mean square of differences between adjacent normal RR intervals, SDNN: standard deviation of all normal RR intervals) and frequency domain (LF: low frequency band ranging from 0.04 to 0.15, HF: high frequency band ranging from 0.15 to 0.4) in absolute units. HRV analysis also included approximate entropy (ApEn) and the Poincaré plot (SD1: standard deviation of the instantaneous variability of the beat-to-beat heart rate, SD2: standard deviation of long-term continuous RR interval variability) [6].

We employed the Kubios^®^ HRV v. 2.0 software to compute these indices [25].

#### The Kupperman-Blatt Menopausal Index (KMI)

This index is composed of 11 sociodemographic and clinical questions to measure the intensity of menopausal symptoms. The index score is categorized into mild (up to 19), moderate (between 20 and 35) and intense (above 35) [26, 27].

#### Study size

The sample power test was calculated from a pilot study according to the RMSSD. The test was based on 42.93±39.33 ms in the TM group and 25.56±14.6 ms in the PM group with 95% confidence level, per alpha risk of 5% and beta of 80%. The sample size determined a required sample of 37 people for each group.

#### Statistical analysis

We completed a Shapiro-Wilk normality test to estimate the distributions. To compare HRV between PM and TM groups we applied non-paired Student t test for parametric distributions and Mann-Whitney test for non-parametric distributions. Statistical significance was considered at the level p<0.05. To evaluate the magnitude of differences between groups we calculated the effect size through Cohen’s *d*. We considered large effect size for values > 0.9, medium effect size for values between 0.9 and 0.5 and small effect size for values between 0.5 and 0.25 [28].

In order to compare HRV categorized based on the magnitude of vasomotor, psychological and somatic symptoms, we applied two way ANOVA followed by Bonferroni post test.

A linear regression analysis was performed in the reverse mode to determine which independent variables (age, transition to menopause, comorbidities, linear indices in the time domain and frequency) influenced the dependent variable (KMI).

## Results

The sociodemographic and clinical profile of the 109 women evaluated are shown in Table 1.

**Table 1:** Socioeconomic, demographic and clinical characteristics of elaborated according to groups menopausal transition and postmenopausal.

Variables	Transition to menopause (n:71)			Postmenopausal (n:38)		
	**N**	**Mean**	**SD**	**N**	**Mean**	**SD**
Age (years)*	71	45.27	3.59	38	54.16	4.52
**Age of menarche (years)**	71	13.27	1.36	38	13.29	1.64
**Age of menopause (years)**	-	-	-	38	47.39	5.69
**Body mass index (BMI)**	71	28.46	5.03	38	28.31	4.95
**Abdominal circumference (cm)**	71	88.23	14.26	38	90.18	13.48
**Systolic arterial pressure (mmHg)**	71	122.18	14.74	38	124.00	14.57
**Diastolic arterial pressure (mmHg)**	71	84.37	16.62	38	84.47	17.66
		**N**	**%**		**N**	**%**
**Race**						
White		10	14.1		6	15.8
Non-white		61	85.9		32	84.2
Marital status*						
Single / widow / divorced		34	47.9		26	68.4
Stable union		37	52.1		12	31.6
**Income** ^**a**^						
No income / less than 1 MW		41	57.7		18	47.4
1–2 MW		28	39.4		20	52.6
≥ 2.1 MW		2	2.8		0	0.0
**Schooling**						
≤ 8 years		45	63.4		30	78.9
> 8 years		26	36.6		8	21.1
**Occupation**						
Employee		20	28.2		12	31.6
Not employee		51	71.8		26	68.4
**Physical activity**						
Yes		17	23.9		9	23.7
No		54	76.1		29	76.3
**Parity**						
0–3		45	63.4		19	50.0
≥ 4		26	36.6		19	50.0
Comorbidity *						
Yes		6	8.5		9	23.7
No		65	91.5		29	76.3

Table 2 presents the KMI questionnaire elaborated with regard to its various components, emphasizing upon the intensity of symptoms under vasomotor, somatic and psychological symptoms, for both groups separately. The women showed moderate menopausal symptoms in both groups with KMI score 23,47 (11,76) at menopausal transition group and KMI score 27,68 (18,57) at postmenopausal group (C = 0,282). Vasomotor symptoms are prominent in the menopausal transition group (C = 0,007) with similar number of hot flushes (C = 0,916).

**Table 2:** Menopause symptoms and Kupperman-Blatt menopausal index (KMI) elaborated according to groups menopausal transition and postmenopausal.

Symptoms	Menopause transition (n:71)		Post-menopause (n:38)		
	**Mean**	**SD**	**Mean**	**SD**	**C**
**Vasomotor**	5.01	4.36	7.26	4.63	0.007
**Paraesthesia**	2.39	1.90	2.47	2.10	0.039
**Insomnia**	2.96	2.33	3.58	2.37	0.263
**Nervousness**	3.18	2.28	2.97	2.39	0.089
**Melancholy**	1.38	1.04	1.53	1.17	0.135
Vertigo*	1.37	1.01	1.63	1.21	0.233
**Weakness**	1.28	1.08	1.24	1.24	0.034
**Arthralgia/Myalgia**	1.65	1.16	1.58	1.28	0.057
**Chronic headache**	2.03	1.06	1.76	1.26	0.231
**Palpitation**	1.48	1.11	1.29	1.11	0.171
**Formication**	1.18	1.16	1.13	1.09	0.044
Number hot flashes per day*	2.85	3.52	3.95	4.24	0.282
**Total KMI score**	23.47	11.76	27.68	18.50	0.916

KMI: Kupperman-Blatt index; C: Cohen’s d

*p<0.05.

Table 3 presents HRV in menopausal transition and postmenopausal women with mild vs. moderate/intense symptoms. We reported that RMSSD, pNN50 and HF band were significantly decreased in the group with moderate/intense symptoms. On the other hand, no significant differences were found for SDNN, LF band and LF/HF ratio.

**Table 3:** HRV in menopause transition and postmenopausal women with mild and moderate/intense symptoms.

HRV indices	Mild (n:48)		Moderate/intense (n:61)		
	**Mean**	**SD**	**Mean**	**SD**	**C**
MeanRR*	901.74	123.25	843.35	122.65	0.47
**SDNN**	27.49	14.18	28.85	11.06	0.1
RMSSD*	31.40	19.40	22.70	12.72	0.53
pNN50*	14.32	15.64	5.80	8.41	0.67
**LFms**^**2**^	351.22	320.96	287.24	293.36	0.2
LFnu*	51.02	16.11	69.44	103.76	0.24
**HFms**^2^*	440.60	530.54	247.08	291.60	0.45
HFnu*	49.52	16.46	42.92	17.72	0.38
**Total**	847.64	839.63	573.64	556.52	0.38
LF/HF*	1.63	1.78	2.02	1.59	0.23

SDNN: Standard deviation of all normal RR intervals; RMSSD: root-mean square of differences between adjacent normal RR intervals; pNN50: percentage of adjacent RR intervals with a difference of duration greater than 50ms; LF: Low frequency; HF: High frequency; ms: milliseconds; mod/int: moderate and intense symptoms; C: Cohen’s d; SD: Standard deviation

*p<0.05.

We divided subjects in menopause transition and post-menopause subjects and compared HRV indices between them. There was significant (small effect size) higher LF (ms^2^) values in the menopause group (Table 4).

**Table 4:** HRV in menopause transition and post-menopause subjects.

HRV indices	Menopause transition (n: 71)		Post-menopause (n:38)		
	Mean	SD	Mean	SD	C
**MeanRR**	857.44	113.37	889.97	146.84	0.24
**SDNN**	26.43	13.25	21.75	11.19	0.38
**RMSSD**	27.72	17.23	24.07	15.13	0.22
**pNN50**	10.05	13.05	8.55	12.53	0.11
**LFms**^2^*	357.04	313.48	233.13	281.03	0.41
**LFnu**	65.32	97.19	53.60	18.84	0.16
**HFms**^**2**^	371.95	471.88	261.73	307.28	0.27
**HFnu**	45.92	16.59	46.27	18.82	0.01
**Total**	775.53	778.44	539.21	526.53	0.35
**LF/HF**	1.96	1.88	1.57	1.16	0.24

SDNN: Standard deviation of all normal RR intervals; RMSSD: root-mean square of differences between adjacent normal RR intervals; pNN50: percentage of adjacent RR intervals with a difference of duration greater than 50ms; LF: Low frequency; HF: High frequency; ms: milliseconds; C: Cohen’s d; SD: Standard deviation

*p<0.05.

HRV was also categorizing based on the magnitude of vasomotor, psychological and somatic symptoms. In Table 5 we noted reduced values of SDNN, LFms^2^, HFms^2^, total power and LF/HF ratio in the somatic symptom compared to vasomotor and psychologic symptoms.

**Table 5:** HRV categorizing based on the magnitude of vasomotor, psychological and somatic symptoms.

HRV indices	Vasomotor		Somatic		Psychologic	
	Mean	SD	Mean	SD	Mean	SD
**MeanRR**	900.84	119.94	931.25	259.72	904.88	168.60
**SDNN**	26.25*	15.02	18.10	15.69	27.73*	16.30
**RMSSD**	29.82	20.71	25.25	22.69	28.46	16.64
**NN50**	171.63	240.85	128.00	181.01	171.00	182.23
**pNN50**	12.20	16.03	10.15	14.35	13.10	13.40
**LFms**^**2**^	332.73*	337.37	125.00	162.63	476.22*	454.84
**HFms**^**2**^	416.66*	587.58	220.50	280.71	396.77*	428.20
**Total**	776.72*	932.85	361.35	463.86	942.33*	927.39
**LF/HF**	1.68*	1.88	0.504	0.097	1.32*	0.60

SDNN: Standard deviation of all normal RR intervals; RMSSD: root-mean square of differences between adjacent normal RR intervals; pNN50: percentage of adjacent RR intervals with a difference of duration greater than 50ms; LF: Low frequency; HF: High frequency; ms: milliseconds; SD: Standard deviation.

*p<0.05 vs. Somatic.

The interference analysis is demonstrated in Table 6 through a linear regression model between menopausal symptoms and independent variables. Regression analysis revealed significant interaction of menopause symptoms with SDNN, RMSSD, pNN50, HF and LF indices. It was verified that the model covered 21.4% of the factors that influence the KMI.

**Table 6:** Influence of age, menopausal symptoms and life span of women in the menopause transition and postmenopausal phase.

	Variable	β	CI (95%)	p	r^2^ adjusted
	Input model*				
	Age (years)	-0.273	-0.969 a 0.422	0.437	0.229
	Transition to menopause	-7.647	-15.794 a 0.499	0.065	
	Comorbities	-5.31	-13.475 a 2.856	0.2	
	SDNN (ms)	1.957	0.613 a 3.302	0.005	
	RMSSD (ms)	-1.182	-2.077 a -0.287	0.01	
	pNN50 (%)	-0.649	-1.211 a -0.087	0.024	
	LF (ms^2^)	-0.03	-0.058 a -0.003	0.03	
	HF (ms^2^)	0.017	-0.001 a 0.035	0.069	
	LF/HF (-)	-0.381	-2.143 a 1.382	0.669	
**Final Model**					
	Transition to menopause	-6.102	-11.626 a -0.578	0.031	0.214
	SDNN (ms)	2.069	0.747 a 3.391	0.002	
	RMSSD (ms)	-1.248	-2.096 a -0.401	0.004	
	pNN50 (%)	-0.615	-1.165 a -0.065	0.029	
	LF (ms^2^)	-0.035	-0.061 a -0.008	0.011	
	HF (ms^2^)	0.018	0 a 0.035	0.049	

* Reverse-mode linear regression analysis between the KMI and independent variables

SDNN: Standard deviation of all normal RR intervals; rMSSD: root-mean square of differences between adjacent normal RR intervals; pNN50: percentage of adjacent RR intervals with a difference of duration greater than 50ms; LF: Low frequency; HF: High frequency.

## Discussion

This investigation was undertaken to evaluate HRV in menopausal transition and post-menopausal symptoms with different intensities. We reported that: 1) menopausal transition and post-menopausal women with more intense symptoms showed reduced HRV; 2) somatic symptoms are suggested to be the main variable related to HRV impairment; 3) time and frequency domain HRV indices presented significant interaction with menopausal symptoms.

Regardless of the menopausal status, the number of hotflushes and the intensity of menopausal symptoms did not show statistical significance. Both groups present moderate to severe menopausal symptoms, and these findings are in agreement with other studies [29–31].

HRV indices represents cardiac autonomic behavior and have been related to menopausal symptoms, mainly vasomotor symptoms and their imbalance to cardiovascular risk [29–31]. In the literature, it is not clear which index represents this imbalance and the involvement of the menopausal state.

We reported significant increased values of RMSSD, pNN50 and HF in the mild symptom group compared to the moderate/intense symptoms group. This result evidences the mixed representation of the sympathetic and parasympathetic autonomic nervous system [12] during this period of the woman's life. The linear regression analysis showed that the lower the HRV indices the higher the symptoms, suggesting vagal withdrawal and concomitant sympathetic activation in women with more intense symptoms. Although previous studies do not support the LF for autonomic regulation, other evidences provide support for this HRV index [11, 32–34].

Previous studies indicated sympathetic activation during hot flushes [29, 30]. Vagal afferent stimulation leads to reflex of vagal efferent excitation and sympathetic efferent inhibition [35]. Attributing this fact only to the LF renders ambiguous interpretation, since this linear index represents a mixture of sympathetic and parasympathetic control, evidencing a possible parasympathetic withdrawal and concomitant sympathetic activation, besides the description of its relation with the baroreflex function [33, 34].

The clinical representation of the LF index arouses controversial interest according to previous evidences [11, 32]. Some authors suggest that it represents a sympathetic activation index [15, 36] however, others described that LF reflects a complex interaction between sympathetic and parasympathetic functions. Other factors such as baroreflex control and direct central rhythmic influences [33, 35] are still not totally identified at low frequency.

The LF/HF ratio is debatable because it does not precisely quantify the sympathovagal balance, in relation to the health-disease process [33, 34] in cardiovascular disease. It is known that parasympathetic activation represents at least 50% of LF and sympathetic activity approximately 25% and the LF is influenced by the mechanical effects of breath [34]. In our study, the LF/HF ratio remained similar regardless of menopausal status.

The association between HRV and the intensity of menopausal symptoms in our study expressed the parasympathetic behavior in the RMSSD and pNN50 indices of HRV, where the higher the intensity of menopausal symptoms the lower the parasympathetic influence, suggesting a decrease in vagal tone with increasing symptom intensity. This results is in agreement with Thurston et al. [16, 17] and De Zambotti et al. [13], which demonstrated a reduction in vagal activity associated with hot flushes, the main menopausal symptom.

Time domain indices are evaluated with statistical calculations of RR intervals and measure the individual cardiac cycle length dispersion around its mean, including the SDNN. This index is considered a marker of the total power (variance) of the HRV and expresses the long-term component responsible for the variability in the period of registration of the HR. Therefore, it is a global index that reflects both sympathetic and parasympathetic modulation [6, 8]. In our study, the SDNN index maintained a proportional relation to the intensity of menopausal symptoms.

In the frequency domain, the HF was increased in women with mild symptoms compared to moderate/intense symptoms, representing higher parasympathetic control in the mild symptoms groups. These results contradict the findings support Thurston et al. [16] and De Zambotti et al. [13] who recorded acute decreases in HF associated with hot flushes, compatible with vagal withdrawal. Moreover, when we evaluated the RMSSD and PNN50 indices, which represent the parasympathetic HR modulation, they were also decreased in women with moderate/intense symptoms and expressed a significant decrease in the parasympathetic predominance with the increase in the intensity of the vasomotor symptoms (presence and frequency of the hot flushes).

The HRV analysis reflects prediction of the internal functions of the body, both in normal and pathological conditions, identifying physiological changes in the organism and the presence of diseases [8, 37]. The action of the ANS on the physiological mechanism of vasomotor symptoms is pertinent thematic, since its neuroendocrine and metabolic functioning is still uncertain scientific literature [14, 38].

In this context, when categorizing based on the magnitude of vasomotor, psychological and somatic symptoms in order to better delineate the role of autonomic modulation, we suggest that somatic symptoms have higher impact on HRV. With this in mind, previous studies have already raised the association between somatic disorders and HRV [39, 40]. Taken together, the literature support the involvement of somatic symptoms in reduced HRV in post-menopausal women.

The limitations of the study consist in a transversal design and can not establish cause and effect relationships with vasomotor symptoms and HRV. The age group is significantly different between two groups and HRV is an age dependent parameter [41]. However, age is a variable related to menopause transition, which does not make possible to provide subjects with similar ages in similar menopause period. In addition, instruments that measure menopausal symptoms, such as KMI, are composed of somatic and psychological clinical manifestations, based on reports. In this way, the physiological reliability of measurement instruments is debatable, since all are passive of failures because it contains a certain margin of error, even after reliability and validity tests.

Our results provide relevant information for the clinical team that deal with menopausal transition and postmenopausal women. Understanding that HRV helps to distinguish menopausal symptoms will aid to plan treatments for this specific population.

## Conclusion

We evidenced that HRV was decreased in menopausal transition and post-menopausal women with more intense symptoms. The findings suggest that somatic symptoms have a close relationship with HRV. Moreover, HRV presented significant association with menopausal transition and post-menopausal symptoms. Our data suggest that HRV may be able to detect the intensity of symptoms menopausal and menopausal transition.

## Supporting information

S1 Data Excel raw data. (XLSX)
