# Correction: Heart rate variability helps to distinguish the intensity of menopausal symptoms: A prospective, observational and transversal study

**DOI:** 10.1371/journal.pone.0229094

**Published:** 2020-02-06

**Authors:** Patrícia Merly Martinelli, Isabel Cristina Esposito Sorpreso, Rodrigo Daminello Raimundo, Osvaldo de Souza Leal Junior, Juliana Zangirolami-Raimundo, Marcos Venicius Malveira de Lima, Andrés Pérez-Riera, Valdelias Xavier Pereira, Khalifa Elmusharaf, Vitor E. Valenti, Luiz Carlos de Abreu

In the Funding section, the following information is missing: This study was supported by a grant under Institutional Agreement number 007/2015 between Faculdade de Medicina do ABC and Secretaria de Saúde do Estado do Acre.

The citation style of the eleventh author is incorrect on PubMed. The correct name is: Abreu LC.

There are errors in references 6, 8–10, 12, 15, 26, 28, 32, 36, and 38. The correct references are:

6. Camm AJ, Malik M, Bigger JT, Breithardt G, Cerutti S, Cohen RJ. Task Force of the European Society of Cardiology and the North American Society of Pacing and Electrophysiology. Heart rate variability: standards of measurement, physiological interpretation and clinical use. Circulation. 1996; 93:1043 65. PMID: 8598068.

8. Vanderlei LCM, Pastre CM, Hoshi RA. Basic notions of heart rate variability and its clinical applicability. Braz J Cardiovasc Surg. 2009; 24:205–217. http://dx.doi.org/10.1590/S0102-76382009000200018.

9. Xhyheri B, Manfrini O, Mazzolini M. Heart rate variability today. Prog Cardiov Dis. 2012; 55:321–31. http://10.1016/j.pcad.2012.09.001.

10. Valenti VE. The recent use of heart rate variability for research. J Hum Growth Develop. 2015; 25:137–140. https://doi.org/10.7322/jhgd.102991

12. Akiyoshi M, Kato K, Owa Y. Relationship between estrogen, vasomotor symptoms, and heart rate variability in climacteric women. J Med Dent Science. 2011; 58: 49–59. PMID: 23896786.

15. Neufeld IW, Kiselev AR, Karavaev AS. Autonomic control of cardiovascular system in pre- and postmenopausal women: a cross-sectional study. J Turk Geriontol Gynecol Ass. 2015; 16:11–20. http://10.5152/jtgga.2015.15201. eCollection 2015.

26. Tarvainen MP, Niskanen JA, Lipponen PO, Ranta-aho Karjalainen PA. Kubios HRV–A software for advanced heart rate variability analysis. Berlin: Springer: In: 4th European Conference os the International Federation for Medical and Biological Engineering. Sloten JV, Verdonck P, Nyssen M, Haueisen J, editors.; 1022–1025, 2008. http://10.1016/j.cmpb.2013.07.024.

28. Sousa RL, Sousa ESS, Silva JCB, Filizola RG. Test-retest reliability in the application of the Menopausal Index of Blatt and Kupperman. Rev Bras Gin Obst. 2000; 22:481–487. http://dx.doi.org/10.1590/S0100-72032000000800003.

32. Lee JO, Kang SG, Kim SH, Park SJ, Song SW. The Relationship between Menopausal Symptoms and Heart Rate Variability in Middle Aged Women. Kor J Fam Med. 2011; 32:299–305. http://10.4082/kjfm.2011.32.5.299.

36. Randall DC, Brown DR, Raisch RM, Yingling JD, Randall WC. SA nodal parasympathectomy delineates autonomic control of heart rate power spectrum. Am J Physiol. 1991; 260:985–988. http://10.1152/ajpheart.1991.260.3.H985.

38. Raimundo RD, Godleski J. Heart Rate variability in metabolic syndrome. J Hum Growth Develop. 2015; 25:7–10. http://dx.doi.org/10.7322/JHGD.96757.

## References

[1] Martinelli PM, Sorpreso ICE, Raimundo RD, Junior OdSL, Zangirolami-Raimundo J, Malveira de Lima MV, et al (2020) Heart rate variability helps to distinguish the intensity of menopausal symptoms: A prospective, observational and transversal study. PLoS ONE 15(1): e0225866 10.1371/journal.pone.0225866 31940354 PMC6961890

